# Supplementary material for: Risk of Hepatocellular Carcinoma in Patients with Porphyria: A Systematic Review
Source: Cancers (Basel). 2022 Jun 15;14(12):2947. doi: 10.3390/cancers14122947 (PMC9221430; doi:10.3390/cancers14122947)
Supplement: Supplementary file 1 [file cancers-14-02947-s001.zip › cancers-1720375-supplementary.pdf]

cancers MDPI

Review

Risk of Hepatocellular Carcinoma in Patients with Porphyria: A Systematic Review

Daryl Ramai, Smit S. Deliwala, Saurabh Chandan, Janice Lester, Jameel Singh, Jayanta Samanta, Sara di Nunzio, Fabio Perversi, Francesca Cappellini, Aashni Shah, Michele Ghidini, Rodolfo Sacco, Antonio Facciorusso and Luca Giacomelli

Table S1. PRISMA checklist.

Section/topic	#	Checklist Item	Reported on page #
		**TITLE**	
Title	1	Identify the report as a systematic review, meta-analysis, or both.	1
		**ABSTRACT**	
Structured summary	2	Provide a structured summary including, as applicable: background; objectives; data sources; study eligibility criteria, participants, and interventions; study appraisal and synthesis methods; results; limitations; conclusions and implications of key findings; systematic review registration number.	3
		**INTRODUCTION**	
Rationale	3	Describe the rationale for the review in the context of what is already known.	4
Objectives	4	Provide an explicit statement of questions being addressed with reference to participants, interventions, comparisons, outcomes, and study design (PICOS).	5
		**METHODS**	
Protocol and registration	5	Indicate if a review protocol exists, if and where it can be accessed (e.g., Web address), and, if available, provide registration information including registration number.	Not registered
Eligibility criteria	6	Specify study characteristics (e.g., PICOS, length of follow-up) and report characteristics (e.g., years considered, language, publication status) used as criteria for eligibility, giving rationale.	6
Information sources	7	Describe all information sources (e.g., databases with dates of coverage, contact with study authors to identify additional studies) in the search and date last searched.	5–6
Search	8	Present full electronic search strategy for at least one database, including any limits used, such that it could be repeated.	5–6 and supplementary table 2
Study selection	9	State the process for selecting studies (i.e., screening, eligibility, included in systematic review, and, if applicable, included in the meta-analysis).	6
Data collection process	10	Describe method of data extraction from reports (e.g., piloted forms, independently, in duplicate) and any processes for obtaining and confirming data from investigators.	6
Data items	11	List and define all variables for which data were sought (e.g., PICOS, funding sources) and any assumptions and simplifications made.	5–6
Risk of bias in individual studies	12	Describe methods used for assessing risk of bias of individual studies (including specification of whether this was done at the study or outcome level), and how this information is to be used in any data synthesis.	5–6
Summary measures	13	State the principal summary measures (e.g., risk ratio, difference in means).	5–6
Synthesis of results	14	Describe the methods of handling data and combining results of studies, if done, including measures of consistency (e.g., I^2) for each meta-analysis.	6
**Section/topic**	**#**	**Checklist item**	**Reported on page #**
Risk of bias across studies	15	Specify any assessment of risk of bias that may affect the cumulative evidence (e.g., publication bias, selective reporting within studies).	9 and supplementary table 3
Additional analyses	16	Describe methods of additional analyses (e.g., sensitivity or subgroup analyses, meta-regression), if done, indicating which were pre-specified.	N/A
		**RESULTS**	
Study selection	17	Give numbers of studies screened, assessed for eligibility, and included in the review, with reasons for exclusions at each stage, ideally with a flow diagram.	7-9
Study characteristics	18	For each study, present characteristics for which data were extracted (e.g., study size, PICOS, follow-up period) and provide the citations.	7–9
Risk of bias within studies	19	Present data on risk of bias of each study and, if available, any outcome level assessment (see item 12).	7–9

Results of individual studies	20	For all outcomes considered (benefits or harms), present, for each study: (a) simple summary data for each intervention group (b) effect estimates and confidence intervals, ideally with a forest plot.	7–9
Synthesis of results	21	Present results of each meta-analysis done, including confidence intervals and measures of consistency.	N/A
Risk of bias across studies	22	Present results of any assessment of risk of bias across studies (see Item 15).	See item 15
Additional analysis	23	Give results of additional analyses, if done (e.g., sensitivity or subgroup analyses, meta-regression [see Item 16]).	N/A
		**DISCUSSION**	
Summary of evidence	24	Summarize the main findings including the strength of evidence for each main outcome; consider their relevance to key groups (e.g., healthcare providers, users, and policy makers).	10–11
Limitations	25	Discuss limitations at study and outcome level (e.g., risk of bias), and at review-level (e.g., incomplete retrieval of identified research, reporting bias).	11–12
Conclusions	26	Provide a general interpretation of the results in the context of other evidence, and implications for future research.	12
		**FUNDING**	
Funding	27	Describe sources of funding for the systematic review and other support (e.g., supply of data); role of funders for the systematic review.	2 (None)

From: Moher D, Liberati A, Tetzlaff J, Altman DG, The PRISMA Group (2009). Preferred Reporting Items for Systematic Reviews and Meta-Analyses: The PRISMA Statement. PLoS Med 6(7): e1000097. doi:10.1371/journal.pmed1000097 For more information, visit: www.prisma-statement.org.

Table S2. Search Strategy.

PubMed

"carcinoma, hepatocellular" [mesh] OR "liver cell carcinoma" OR "hepatic cell carcinoma" OR "hepatocellular carcinoma" OR "hepatic carcinoma" OR "hepatocarcinoma" OR "hepatoma" OR "liver carcinoma" OR "liver neoplasms" [mesh] OR "HCC" OR "liver cancer"

"porphyrias" [mesh] OR "porphyria" OR "porphyrias" OR "porphyric disease" OR "porphyric" OR "protoporphyria, erythropoietic" [mesh]

Embase

"liver cell carcinoma"/exp OR "carcinoma, hepatic cell" OR "carcinoma, hepatocellular" OR "carcinoma, liver" OR "carcinoma, liver cell" OR "hepatic carcinoma" OR "hepatic cell carcinoma" OR "hepatocarcinoma" OR "hepatocellular carcinoma" OR "hepatoma" OR "liver carcinoma" OR "liver carcinoma rupture" OR "liver cell carcinoma" OR "malignant hepatoma" OR "primary liver carcinoma" or "liver tumor"/exp

"porphyria"/exp OR "intermittent porphyria" OR "latent porphyria" OR "porphyria" OR "porphyria, latent" OR "porphyrias" OR "porphyric disease" OR "systemic porphyria" not porphyrin*

Web of Science

"Hepatocellular carcinoma" OR "hepatic cell carcinoma" OR "hepatic carcinoma" OR "hepatocarcinoma" OR "liver cancer*" OR "HCC" OR "hepatic neoplasia" OR "hepatic neoplasias"

AND

porphyria* or porphyric

Table S3. Quality Assessment using the Newcastle-Ottawa Scale (NOS).

Study	Representativeness	Selection	Ascertainment of exposure	Demonstration that outcome of interest was not present at start of study	Factors comparable between groups?	Assessment of outcome?	Follow-up	Adequacy of follow-ups	Overall Quality Score

	Population based ?	Drawn from same community as exposed cohort	Secured records, clinical outcomes	Not present	Yes?	Independent blind assessment, record linkage	Mentioned or not mentioned	Complete follow-up, or subjects lost to follow-up unlikely to introduce bias	(Maximum = 9)
**Solis 1982 [13]**		★	★	★	★	★	★		**6**
Hardell 1984 [14]		★	★	★	★	★	★		**6**
Lithner 1984 [15]		★	★	★	★	★	★		**6**
Salata 1985 [16]		★	★	★	★	★	★		**6**
Siersema 1992 [17]		★	★	★	★	★	★		**6**
Kauppinen 1992 [11]		★	★	★	★	★	★		**6**
Andersson 1996 [12]		★	★	★	★	★	★		**6**
Linet 1999 [18]		★	★	★	★	★	★		**6**
Andant 2000 [4]		★	★	★	★	★	★	★	**7**
Lissing 2022 [23]		★	★	★	★	★	★		**6**
Fracanzani 2001 [19]		★	★	★	★	★	★		**6**
Gisbert 2004 [20]		★	★	★	★	★	★		**6**
Cassiman 2008 [21]		★	★	★	★	★	★		**6**
Innala 2011 [8]		★	★	★	★	★	★	★	**7**
Sardh 2013 [10]		★	★	★	★	★	★		**6**
Elder 2013 [7]		★	★	★	★	★	★		**6**
Lang 2015 [22]		★	★	★	★	★			**5**
Baravelli 2019 [5]		★	★	★	★	★	★		**6**
Saberi 2020 [6]		★	★	★	★	★	★		**6**
